# Dysbiosis of fish gut microbiota is associated with helminths parasitism rather than exposure to PAHs at environmentally relevant concentrations

**DOI:** 10.1038/s41598-022-15010-2

**Published:** 2022-06-30

**Authors:** Yannick Colin, Noëlie Molbert, Thierry Berthe, Simon Agostini, Fabrice Alliot, Beatriz Decencière, Alexis Millot, Aurélie Goutte, Fabienne Petit

**Affiliations:** 1 CNRS, M2C, UNICAEN, UNIROUEN, Normandie University, 76821 Rouen, France; 2 CNRS, EPHE, UMR METIS, Sorbonne Université, 75005 Paris, France; 3 Département de biologie, Centre de recherche en ecologie expérimentale et prédictive (CEREEP-Ecotron IleDeFrance), Ecole normale supérieure, CNRS, PSL University, 77140 Saint-Pierre-lès-Nemours, France; 4 EPHE, UMR 7619, PSL Research University, Sorbonne University, 4 place Jussieu, 75005 Paris, France

**Keywords:** Parasite host response, Microbiome, Microbiology

## Abstract

Although parasite infection and pollution are common threats facing wild populations, the response of the gut microbiota to the joint impact of these stressors remains largely understudied. Here, we experimentally investigated the effects of exposure to Polycyclic Aromatic Hydrocarbons (PAHs) and infection by a common acanthocephalan intestinal parasite (*Pomphorhynchus sp.*) on the gut microbial flora of a freshwater fish, the European chub (*Squalius cephalus*). Naturally infected or uninfected individuals were exposed to PAHs at environmentally realistic concentrations over a five-week period. Characterization of the gut bacterial community through 16S rRNA gene amplicon sequencing revealed that parasitic infection was a more structuring factor of bacterial diversity and composition than PAH exposure. Specifically, chub infected by *Pomphorhynchus sp.* harbored significantly less evenly represented gut bacterial communities than the uninfected ones. In addition, substantial changes in sequence abundance were observed within the main bacterial phyla, including the *Firmicutes*, *Fusobacteriota*, *Actinobacteriota*, and *Proteobacteria*. Again, these compositional changes correlated with host infection with *Pomphorhynchus sp.*, confirming its pivotal role in gut microbial assemblage. Overall, these results highlight the importance of defining the parasitic status of individuals when conducting microbial ecotoxicological analyses at the digestive tract level, as this should lead to better understanding of microbiota modulations and help to identify microbial markers specifically associated with chemicals.

## Introduction

In the last few decades, chemical contamination of natural systems has become an increasing threat to animals and their related microbiota. Several pollutants such as pesticides^1^, heavy metals^2^, persistent organic pollutants^3^ and antibiotics^4^ can trigger imbalances in the microbial flora, termed “dysbiosis”^5^. Changes in alpha diversity and/or community composition of host-associated gut microbial communities are not innocuous as the gut microbiota fulfills crucial functions, such as the metabolism of nutrients, contaminants and drugs, maintenance of the gut mucosal barrier and regulation of the immune system^5–7^. Gastrointestinal dysbiosis may subsequently increase susceptibility to pathogens or be responsible for inflammatory and metabolic diseases, thereby affecting host fitness^8–10^. For instance, mice treated with cadmium or carbendazim, a fungicide, exhibited altered gut microbiota coupled with impaired energy metabolism, nutrient absorption and immune system function^11,12^.

The gut microbial community is therefore increasingly perceived as a valuable physiological marker of stress, and is included in ecotoxicological studies to draw up a health assessment of organisms, particularly in mammals^13–15^. For this purpose, high-throughput sequencing technologies are now routinely applied to the 16S rRNA gene amplicon to assess changes in host-associated microbial communities facing pollutant exposure. This molecular approach provides a comprehensive view of the microbiota and enables a more or less fine-grained analysis depending on the taxonomic level considered (from phylum to genus). However, meta-analyses conducted on 16S rRNA gene libraries have struggled to define the "normal" composition of the microbiota (i.e., the eubiotic state) due to the influence of factors other than contaminants that also significantly drive gut microbial community assemblage^15^. These additional structuring parameters are not systematically considered or have simply not yet been identified. Hence, the baseline gut microbiota composition of a given host organism may differ from one study to another, which inevitably leads to differential responses to stress and challenges scientists to identify microbial proxies that reflect dysbiosis in response to chemical stress^16^.

In aquatic systems, beyond the impact of chemical contamination, several environmental variables (e.g., water quality, diet, temperature or pH) and intrinsic factors (e.g., sex, age, genotype or developmental stage) have been reported to shape gut microbiota in ectothermic animals such as fish^17–23^. More recently, the occurrence of intestinal parasitic helminths in fish and their interactions with the host and its gut microbiota has gained increasing consideration^24–26^. By inhabiting the same ecological niche, parasites are likely to interact directly with gut microbial communities, for example by secreting peptides with bactericidal or bacteriostatic activities^27,28^. Parasitism can also indirectly impact fish gut microbiota by triggering mechanical damage and inflammation of the gut wall^29^, sometimes reducing the growth and survival rates of the host^30,31^. Ultimately, in some cases, parasites may induce beneficial effects by accumulating pollutants and thus mitigating the impact of pollution on host tissues and presumably on the associated microorganisms^32–34^.

On the basis of these preliminary findings and due to the prevalence and diversity of intestinal parasites, one might expect that the parasitic status would be considered when conducting ecotoxicological studies on fish gut microbiota. However, studies tackling the joint impact of pollution and parasitism on gut microbiota and the consequences for the hosts remain scarce, either in rearing or in natural systems^35^. Those three-way interactions are indeed hard to predict given that parasite and host species, as well as the targeted chemical are expected to influence the findings. To fill this gap, we conducted an experiment on European chub (*Squalius cephalus*) to jointly assess the effect of PAH exposure and infection with *Pomphorhynchus sp.,* a member of the *Acanthocephala* group, on the host gut microbiota. We previously showed that PAHs accumulate in parasites instead of host tissues and that uninfected chub were subjected to higher oxidative stress than infected ones^34^. Here, high-throughput 16S rRNA gene sequencing was applied to assess changes in bacterial alpha-diversity and community composition associated with PAH exposure and/or parasite infection. The objectives of this work were (1) to compare the contribution of environmentally relevant PAH concentrations to that of the helminthic parasite on gut microbial communities, and (2) to determine whether the occurrence of the parasite can mitigate the potential deleterious effects of PAHs on gut microbial communities.

## Results

At the end of the five-week exposure period, 69% of the controls and 67% of the PAH-contaminated fish were found to be infected with the intestinal parasite *Pomphorhynchus sp*. Regarding infected chub, the parasite load ranged from two to nine parasites per individual and the pools of infected chub used for molecular analyses had an average parasite burden of between 3.0 and 6.3 (Table S1). The number of intestinal parasites did not differ among controls and PAH-exposed chub (Mann Whitney Wilcoxon test*, p* > 0.05).

### Processing of 16S rRNA sequencing data

The sequencing of the amplicon libraries resulted in 3,020,539 raw sequences (Table S2). After quality filtering, denoising and merging of paired-end reads, 63.8% of the raw dataset were retained, for a total of 1,898,430 high quality non-chimeric reads with a median of 78,355 reads per sample (Table S2). Overall, 6,706 bacterial Amplicon Sequence Variants (ASVs) were defined, with an average of 1578 ± 509 ASVs per sample. Of these ASVs, 98.1% were classifiable at the phylum level and 78.0% were classifiable at the genus level. Rarefaction curve analyses, which depict the relationship between the number of reads and the number of ASVs detected, were drawn (Fig. S1).

### Alpha bacterial diversity

**Figure 1:**
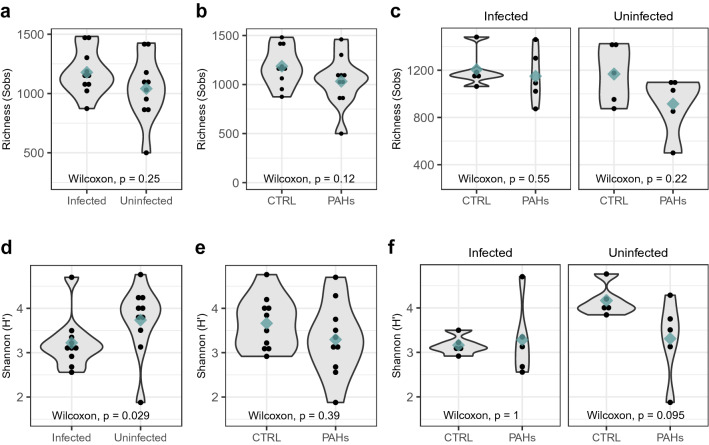
Gut bacterial richness and diversity. Observed richness (S_obs_, panels **a**–**c**) and Shannon–Weaver (H′, panels **d**–**f**) indexes were estimated for fish gut bacterial communities. Alpha-diversity indexes were compared between infected and uninfected chub (n = 20 samples; panels **a** and **d**) and between controls and PAH-exposed chub (n = 20; panels **b** and **e**). In parallel, PAH effect was analyzed independently on uninfected (n = 10) and infected chub (n = 10) (panels **c** and **f**). The median is shown as a green diamond. Mann Whitney Wilcoxon tests were used to investigate differences between groups and the *p* values are shown on the plots.

Alpha bacterial diversity was primarily found to correlate with the host parasite status. The gut bacterial communities of infected chub exhibited significantly lower Shannon values (H′ = 3.2 ± 0.6) compared to those of uninfected chub (H′ = 3.7 ± 0.8) (Mann Whitney Wilcoxon test*, p* < 0.05) (Fig. 1d, Table S3), while both groups harbored similar bacterial richness (S_obs uninfected_ = 1044 ASVs ± 279 and S_obs infected_ = 1177 ASVs ± 178, Mann Whitney Wilcoxon test, p > 0.05) (Fig. 1a, Table S3). In contrast, no significant change in bacterial diversity indexes was attributed to PAH exposure (*p* > 0.05), although PAH-exposed fish tended to have somewhat less rich (S_obs_ = 1,029 ASVs ± 256) and less diverse (H′ = 3.3 ± 0.8) gut bacterial communities than the controls (S_obs_ = 1,191 ASVs ± 199 and H′ = 3.7 ± 0.6) (Fig. 1b,e, Table S3). To get rid of the parasite effect, Mann Whitney Wilcoxon tests were re-run to assess the impact of PAHs only on chub with the same infection status. As a result, PAHs again showed a non-significant tendency to affect bacterial richness in both uninfected and infected chub (Fig. 1c). Likewise, PAHs were observed to decrease the Shannon diversity index, but this decline was much more marked in uninfected chub (H′_CTRL_ = 4.2 ± 0.4 vs. H′_PAHs_ = 3.3 ± 0.9, *p* = 0.095) than in parasitized fish (H′_CTRL_ = 3.2 ± 0.2 vs. H′_PAHs_ = 3.3 ± 0.8, *p* = 1) (Fig. 1f).

### Bacterial community structure and composition

**Figure 2:**
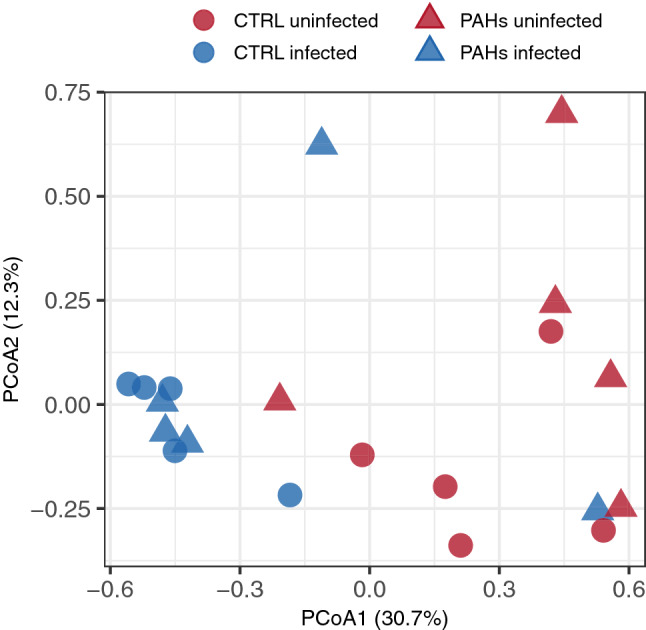
Changes in the fish gut bacterial community composition profiles. The PCoA ordination is based on the Bray–Curtis dissimilarity metric. The shape of the symbols refers to PAH exposure: round for controls and triangular for PAH-exposed fish. The color refers to the parasitic status of the host: red for uninfected chub and blue for infected chub.

A principal coordinate analysis (PCoA) was conducted at the ASV level and showed a clear dichotomy between gut bacterial community profiles according to whether animals were infected or not with *Pomphorhynchus sp.* at the time of the sampling (Fig. 2). On the ordination plot, infected chub were separated from the non-infected ones along the first axis (PCoA1) explaining 30.7% of the total variation. The effect of the parasite on the bacterial community structure was supported by a permutational multivariate analysis of variance (PERMANOVA, *F*_parasite_ = 5.4, *p* < 0.001). Regarding changes in bacterial community structure related to PAH exposure, no clear effect could be evidenced in the multivariate ordination as supported by a PERMANOVA (F_PAHs_ = 1.1, *p* = 0.31) (Fig. 2). Variation partitioning analysis further indicated that parasite occurrence and PAH exposure accounted independently for 17% and 1% of the total variance, respectively. A joint explanatory effect for these two variables was not observed.

**Figure 3:**
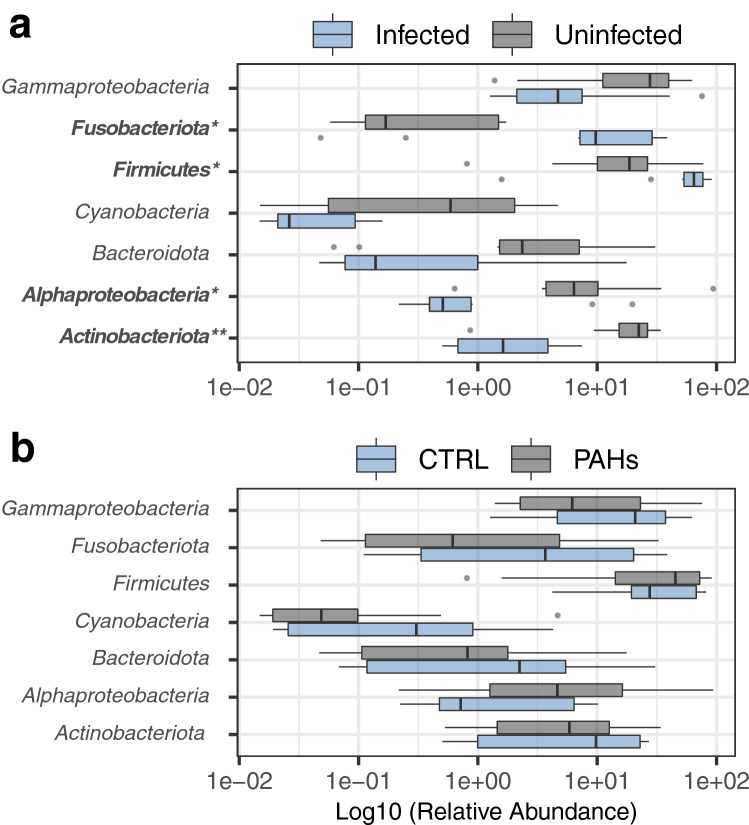
Relative abundance of the main gut bacterial phyla. The relative abundances of the main bacterial phyla were compared between infected and uninfected chub (n = 20 samples) (**a**) and between controls and PAH-exposed chub (n = 20 samples) (**b**). The color refers to the parasitic status (gray: uninfected and blue: infected) or the PAH exposure (gray: PAH-exposed and blue: controls). Mann Whitney Wilcoxon tests were used to compare the groups for each taxon, and significantly differentially abundant taxa were highlighted in bold on the plot. The *p* values were adjusted with Benjamini–Hochberg corrections to correct for multiple testing (*: *p* < 0.05 and **: *p* < 0.01) (see Table S5 for details of adjusted *p* values and Table S4 for average relative abundances per groups).

**Figure 4:**
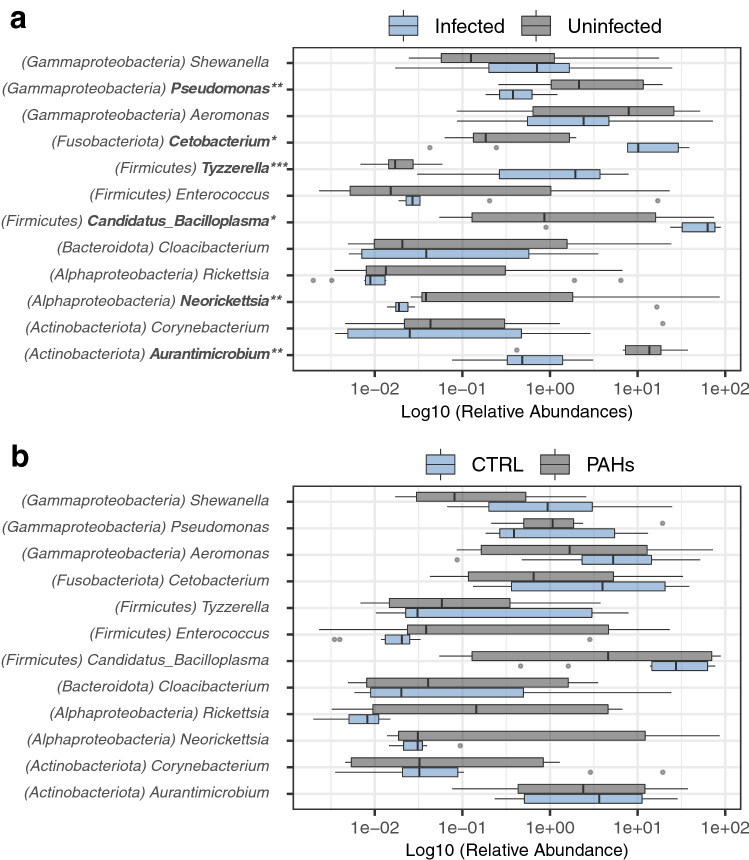
Relative abundance of the main bacterial genera in gut bacterial communities. The relative abundances of the main bacterial genera were compared between infected and uninfected chub (**a**) and between controls and PAH-exposed chub (**b**). The dot color refers to the parasitic status (gray: uninfected and blue: infected) or the PAH exposure (gray: PAH-exposed and blue: controls). Mann Whitney Wilcoxon tests were used to compare the groups for each taxon and significantly differentially abundant taxa were highlighted in bold on the plot. The *p* values were adjusted with Benjamini–Hochberg corrections to correct for multiple testing (*: *p* < 0.05; **: *p* < 0.01 and ***: *p* < 0.001) (see Table S6 for details of adjusted *p* values).

Taxonomic classification of ASVs revealed that most 16S sequences of gut bacterial communities were primarily assigned to the *Firmicutes (41.4% of total reads)*, *Proteobacteria (31.3%; Alpha and Gamma classes)*, *Actinobacteriota (11.4%)*, *Fusobacteriota (8.5%)*, *Bacteroidota (4.5%)* and *Cyanobacteria* (0.7%) (Fig. S2 and Table S4). Significant changes in the relative abundance of these major phyla were recorded among the samples and were associated with the parasitic infection. Specifically, infected chub harbored higher relative abundances of *Firmicutes* (Wilcoxon rank test, *p* < 0.05) and *Fusobacteriota* (*p* < 0.05) (Fig. 3a and Table S5). In particular, the genera *Candidatus Bacilloplasma* (425 ASVs)*, Tyzzerella* (92 ASVs) and *Cetobacterium* (677 ASVs) were significantly enriched in infected chub (*p* < 0.05) (Fig. 4a and Table S6). The presence of the parasite was further correlated with significantly lower proportions of the *Actinobacteriota* (*p* < 0.01) and *Alphaproteobacteria* (*p* < 0.05) (Fig. 3a and Table S5). At the genus level, the *Aurantimicrobium* (*p* < 0.01)*, Cutibacterium* (*p* < 0.05)*, Neorickettsia* (*p* < 0.01) and *Pseudomonas* (*p* < 0.01) were observed to decrease in relative abundance in infected chub (Fig. 4a and Table S6).

Conversely, none of the bacterial taxa differed significantly between controls and PAH-exposed individuals at either the phylum (Fig. 3b and Table S5) or genus levels (Fig. 4b and Table S6). However, some non-significant trends could still be observed at the genus level, such as a decrease in the abundance of sequences related to *Shewanella* and an increase in the abundance of sequences related to *Enterococcus* and *Rickettsia* in PAH-exposed chub*.* It was unfortunately not possible to achieve more significant results by analyzing infected and uninfected fish separately (Figs. S3 and S4; Tables S7 and S8), although it appears that differences between controls and PAH-exposed chub were more consistent in the uninfected chub.

## Discussion

Persistent organic pollutants such as PAHs can lead to gut microbiota dysbiosis and multiple potential adverse effects for the host organisms^3,36^. Therefore, the monitoring of the microbial communities in the gut of aquatic species as sentinel organisms may help to indicate habitat health condition. However, gut microbiota is concurrently influenced by many other biotic and abiotic parameters. In particular, we hypothesized that the presence of helminthic parasites in the digestive tract of host organisms might be a key factor to consider, as parasites are likely to interact physically with the gut microbiota^25,26^ but also mitigate the impact of pollutants on the host and its microbiota thanks to their bioaccumulative capacities^33,37^. Based on the same experiment, quantification of reactive oxygen metabolites in chub plasma suggested that oxidative stress induced by PAH exposure was reduced in individuals infected with *Pomphorhynchus sp.*^38^. These multi-faceted interactions between the host, its microbiota and contaminants are likely to blur our understanding of the microbial changes actually driven by pollutants and, if neglected, may compromise our assessment of the risks associated with environmental contamination. In this context, this work aimed at addressing the joint effect of environmentally relevant PAH concentrations and parasitism on the gut microbiota in wild European chub and to test whether an interaction between these two structuring factors occurs.

Compared to PAH exposure, parasitism had a more significant impact on the gut bacterial diversity. Chub infected with *Pomphorhynchus sp.* harbored significantly less diverse bacterial communities than uninfected fish. This finding suggests that fish harbor less evenly distributed gut bacterial taxa when parasitized*.* It is difficult to determine whether reduced bacterial diversity is a cause or consequence of parasitic infection. Indeed, it may be assumed that hosts with lower bacterial diversity might be more susceptible to parasite infection^39^. Alternatively, parasites that are able to colonize the intestinal tract may impair microbial communities^26^ through damage to the gut epithelium or overlapping resource requirements^40^. Interestingly, treatment and suppression of parasitic infection in mice resulted in partial restoration of the gut microbiota, providing evidence for the responsibility of parasitic infection in microbial dysbiosis^41^. Although negative and positive effects of parasite infection on gut microbiota have been reported in the literature, there is no clear consensus about the direction of the effect^24,26^. In fact, given the complexity of these three-way relationships, results are contingent on the studied systems^26,39,42^.

When comparing individuals with similar infection status to remove the parasite effect, PAH exposure resulted in only moderate and non-significant changes in alpha diversity indexes, namely, uninfected chub exposed to PAHs showed a slight decrease in the number of ASVs while infected chub maintained a bacterial richness similar to controls. Regarding the Shannon-Weiner index, gut bacterial diversity also tended to decrease in individuals exposed to PAHs, particularly in chub that were not infected with *Pomphorhynchus sp*. Such a result does not seem to reflect any protective effect of the parasite toward PAHs, but is rather due to the lower Shannon index recorded in infected chub. The decrease in alpha diversity indexes is consistent with previous studies showing that gut microbial diversity of freshwater fish was inversely correlated with PAHs levels in the muscles of wild fish exposed to an oil spill^43^ or with the dietary exposure level of juveniles to benzo[a]pyrene^44^. Similar observations were made in marine systems, where exposure to crude oil or PAH-contaminated sediments was associated with a loss of gut microbial diversity^3,45^.

In accordance with previous studies on freshwater fish^24,46,47^, the gut microbiota of chub was characterized by the prevalence of the phylum *Proteobacteria*, *Firmicutes*, *Actinobacteriota*, and *Fusobacteriota*. Significant variations in bacterial community composition occurred between the samples both at the phylum and genus levels, but changes were not attributable to PAH contamination. This lack of microbial response to this contaminant was unexpected, given that PAHs and their metabolites were detected in the liver and muscle and that individuals experienced liver hypertrophy^38^. It was also not in line with a number of studies that evidenced an effect of PAHs, sometimes even characterized by the recruitment of hydrocarbonoclastic bacteria^3,43,44,48–50^. This discrepancy may stem from the fact that the PAH concentration tested in this work is much lower than those generally applied in mesocosm studies or recorded during acute pollution episodes. Moreover, although the concentration of PAHs tested in this work is consistent with the level of contamination faced by wild fish populations in urbanized watersheds, our experimental design consisted of exposing individuals to PAHs once a week and not continuously, as evidenced from in situ monitoring. Beyond contaminant concentrations, how fish are exposed to PAHs varies across mesocosm studies, with the contaminant being introduced in water^3,50^, sediment^36,49^ or diet^44^, further impeding comparison of data across studies.

In contrast to PAHs, parasitism was significantly related to an enrichment of *Firmicutes* and *Fusobacteriota*. Specifically, two bacterial genera, namely *Candidatus Bacilloplasma* and *Tyzzerella*, solely explained the increase in abundance of *Firmicutes* in infected fish. *Candidatus Bacilloplasma,* (*Mollicutes* class)*,* was initially identified from the intestinal surface of the isopod *Porcellio scaber*^51^ and later in other crustaceans such as shrimp, lobster or crab^47,52,53^. This taxon, attaching to the tips of the cuticular spines of the gut wall, was previously hypothesized to promote the digestive process in the host and to regulate the expression of immune genes^47^. However, *Candidatus Bacilloplasma* was also repeatedly found to be enriched in shrimp suffering from Acute Hepatopancreatic Necrosis Disease (AHND)^53,54^, their increased abundance being possibly related to the reduction in overall microbial diversity triggered by the disease^54^. The genus *Tyzzerella* has been previously described as being enriched in the gut microbiota of fish with a herbivorous diet^55^. Within the *Fusobacteria*, a significant differential abundance between infected and uninfected chub was mainly attributed to *Cetobacterium*. Members of this genus were recently demonstrated to provide benefits to the host by improving glucose homeostasis in fish^56^. On the other hand, parasitism was associated with a decline of sequences affiliating with the *Actinobacteriota* (*Aurantimicrobium*) and *Proteobacteria* (*Neorickettsia* and *Pseudomonas*). *Actinobacteria* are prolific producers of antimicrobial molecules and were posited as probiotic agents in aquaculture^57^. Nevertheless, due care must be exercised regarding parasite effects since the parasitic status of chub was not manipulated in this experiment. It is indeed conceivable that another unknown confounding factor could explain both (i) whether chub are naturally infected or not with *Pomphorhynchus sp*. and (ii) changes in the gut microbiota, thus rendering the observations previously made between parasite occurrence and shifts in bacterial phyla and genera purely correlational and not indicative of a cause-and-effect relationship. For instance, diet has been reported to influence both bacterial communities^18^ and gastrointestinal parasitic infections^58^, the latter being trophically transmitted. Therefore, the degree to which intestinal parasites altered the gut microbiota of fish in polluted environments needs to be further evaluated, using experimental infections in which infections take place sequentially.

Although PAH exposure induced oxidative stress in chub^38^, this work suggests that the gut microbiota appears to be influenced more by parasitic infection than by chemical contamination when tested at environmentally relevant concentrations. This parasitic effect has important implications for ecotoxicological studies, as examining the response of the fish microbiota while neglecting the presence of intestinal helminths in hosts may lead to an incomplete understanding of the effects of contaminants on aquatic organisms. Because of the absence of a clear effect of PAHs, it was unfortunately not possible to test a potential interaction between the parasite and the pollutant. Although our results provide a general overview of the taxonomic profile of the gut bacterial community of chub exposed to PAHs and parasites, we could not conclude on the fitness consequences of the altered microbiota. Future research should seek to assess the functional impact of host microbiome composition in the face of environmental pollution and parasite infection, especially given that bacterial communities are able to limit absorption of chemicals into the small intestine, biotransform contaminants (e.g., PAHs) and regulate the expression of major detoxification enzymes (CYP450)^59^.

## Methods

Animal care protocols were performed in accordance with laws on animal experimentation in France and Europe, and were approved by the national ethics committee for animal experimentation under file number APAFIS#2018111614171570. Animals were captured and experimented upon authorization 2019-DDT-SSE-37 delivered by the Préfecture de l’Essonne. The authors declare that they have complied with the ARRIVE guidelines.

### Housing conditions, experimental design and sample collection

Wild European chub (*S. cephalus*) were captured on a tributary of the Marne River (49°5′42′′N, 3°40′23′′E) where the levels of chemical contamination have been studied for 35 years within the multidisciplinary research network PIREN Seine (https://www.piren-seine.fr/en), and the prevalence of intestinal parasites *Pomphorhynchus* sp. is known^34^. Chub were electrofished during a period of one week in January 2019 and selected based on specific body length (mean ± SD; 56.7 ± 28.2 g; 16.6 ± 2.67 cm). Visual inspection of the chub did not allow for unambiguous sex determination as the individuals were not yet mature. Individuals were brought back to the CEREEP–Ecotron facilities and placed into outdoor tanks (n = 5; 3 m^3^) under natural temperature conditions for one week. Anesthetized chub (M222, 80 mg.L^−1^) were carefully tagged with a passive integrated transponder device (8 mm × 1.4 mm FDX-B skinny tag, OREGON RFID Portland, USA) inserted intraperitoneally, weighed (± 0.5 g) and measured (± 0.1 cm). Tagged fish were randomly divided into 175 L indoor tanks (80 × 60 × 42 cm). After two weeks of acclimation, chub were randomly divided into two PAH exposure groups (0.1X and 10X), five replicate tanks per group and 10 fish per tank. Additional information about animal husbandry, PAH exposure and monitoring of biological and physicochemical parameters are presented elsewhere^38^. Briefly, a five-week experimental exposure to PAHs was performed with vegetable oil containing a mixed solution of 16 PAHs dissolved in cyclohexane at 10 ng µL^-1^ each, purchased from LGC standards. PAH exposure was performed on sedated fish once a week over a period of five weeks by injecting 1 ml of contaminated oil into the stomach using a syringe fitted with plastic tubing (0.1 × 12 cm, diameter x length). The fish were carefully observed for 10 min after the contamination to control for oil regurgitation, thus the dose of PAHs diluted in vegetable oil was considered the administered dose. The 0.1X concentration (i.e., 50 ng PAHs g^-1^ vegetable oil) is representative of the concentration found in commercial fish pellets used to feed chub during acclimation and experimental activity. Fish exposed to this level of PAHs were used to account for the effect of experimental conditions that may affect the gut bacterial community and hereafter are referred to as control samples. In contrast, the 10X concentration (i.e., 5000 ng PAHs g^-1^ of vegetable oil), corresponds to the level of PAHs quantified in wild chub in the Marne River, France (unpublished data) that flows through an industrial and densely populated basin. The 10X exposure reflects similar concentrations reported in fish from different geographical areas^45,60^. Fish exposed to this contamination level are hereafter referred to as PAH-exposed chub.

Since it was known that about half of the individuals are naturally infected with *Pomphorhynchus sp.*, the experiment was conducted blind to this factor, with the parasite status being determined by dissection only at the end of the study. Following the five-week PAH exposure period, all chub were sacrificed and the intestine and body cavity were examined for the presence of acanthocephalan parasites. The whole intestine of the chub was dissected under sterile conditions and parasites were first removed. Gut microbiota samples were collected by scraping away the intestinal wall with sterile scalpel blades and then frozen at −80 °C until DNA extraction. Note that the samples were free of fecal material as the sampling was conducted prior to feeding. No sample exclusion criteria were defined or applied.

### Total DNA extraction and 16S rRNA gene amplicon high-throughput sequencing

To obtain sufficient biological material for DNA extraction, gut samples of three fish exposed to the same level of PAHs, from the same experimental tank and exhibiting the same parasite status were pooled. A total of 20 sample pools were then used for downstream microbial analysis (i.e., five control-infected, five control-uninfected, five PAH-infected, five PAH-uninfected, Table S1). Total DNA was extracted with the DNeasy PowerSoil Kit (Qiagen) according to the manufacturer’s instructions. Sample biomasses used for DNA extractions are shown in Table S1. DNA concentration and quality were assessed with a Nanodrop spectrophotometer (NanoDrop™ 2000/2000c, Thermo Scientific). The 341F/785R primer set targeting the hypervariable V3-V4 region were used to amplify the bacterial 16S rRNA gene fragment (expected amplicon size ~ 440 bp) (Klindworth et al., 2013). Paired-end sequencing was performed at MrDNA Molecular Research LP (Shallowater; TX, USA) using the Illumina MiSeq workflow.

### Bioinformatics and statistical analyses

Raw 16S rRNA sequencing reads were quality filtered using the dada2 R package (v 1.16.0) and its associated pipeline^61^. Low-quality base pairs were removed using the ‘filterAndTrim’ function by setting the maximum expected errors parameter (maxEE) to 2 for both paired reads and truncating the forward and reverse reads at 250 bp and 210 bp, respectively. Exact biological sequences present in the sample (ASVs) were defined and taxonomies were assigned in accordance with the dada2 pipeline from the SILVA rRNA reference database (v.138) using the Ribosomal Database Project naive Bayesian classifier method^62^.

All subsequent analyses were then carried out in the R environment (v.4.0.3). The ASVs accumulation curves were drawn using the ‘rarecurve’ function in the vegan package (v.2.5.7)^63^. Observed richness and Shannon diversity indexes were calculated using the ‘estimate_richness’ function in the phyloseq package (v.1.32.0)^64^. Alpha diversity analyses were based on a rarefied dataset (30,845 reads per sample) in order to remove biases linked to uneven sequencing depth. Since our data were not normally distributed, nonparametric tests were applied. The significance of the changes in the alpha diversity parameters were tested using Mann Whitney Wilcoxon tests and the ‘stat_compare_means’ function in the ggpubr package (v.0.2.4). Bray–Curtis distances calculated on Hellinger-transformed data were used to determine shifts in the gut bacterial community using a PCoA ordination. The effect of PAH exposure and parasite infection were tested using permutational analyses of variance (PERMANOVA) with the vegan package. The function ‘varpart’ from the vegan package was used to quantify the relative, joint, and unique contribution of predictor variables to explain bacterial community compositional variations between groups. Changes in bacterial community composition were further explored at the phylum and genus levels. Differentially abundant bacterial taxa between test groups were identified using Mann Whitney Wilcoxon tests. To correct for multiple comparisons, a Benjamini–Hochberg False Discovery Rate adjustment was used (cut-off value: 0.05).

## Supplementary Information

Supplementary Information.

## Data Availability

The raw 16S rRNA gene sequences generated during this study were deposited in the European Nucleotide Archive (http://www.ebi.ac.uk/ena/data/view/PRJEB48108). The R code used to process the amplicon sequences, generate the phyloseq object, and produce all the figures, tables and statistical analyses is available from Github at the URL https://github.com/ycolin26/chub_pahs_parasite.
