# Supplementary material for: Dysbiosis of fish gut microbiota is associated with helminths parasitism rather than exposure to PAHs at environmentally relevant concentrations
Source: Sci Rep. 2022 Jun 30;12:11084. doi: 10.1038/s41598-022-15010-2 (PMC9246949; doi:10.1038/s41598-022-15010-2)
Supplement: Supplementary file 1 — Supplementary Information. [file 41598_2022_15010_MOESM1_ESM.docx]

**TITLE: Dysbiosis of fish gut microbiota is associated with helminths parasitism rather than exposure to PAHs at environmentally relevant concentrations**

Yannick Colin^1,2*^, Noëlie Molbert^2*^, Thierry Berthe^1,2^ Simon Agostini^3^, Fabrice Alliot^2,4^, Beatriz Decencière^3^, Alexis Millot^3^, Aurélie Goutte^2,4^ and Fabienne Petit^1,2^

^1^UNIROUEN, UNICAEN, CNRS, M2C, Normandie University, 76821 Rouen, France

^2^Sorbonne Université, CNRS, EPHE, UMR METIS, F-75005, Paris, France

^3^Centre de recherche en écologie expérimentale et prédictive (CEREEP-Ecotron IleDeFrance), Département de biologie, Ecole normale supérieure, CNRS, PSL University, 77140 Saint-Pierre-lès-Nemours, France

^4^EPHE, PSL Research University, UMR 7619 Sorbonne University, F-75005, 4 place Jussieu, Paris, France

* These authors contributed equally to this study

**Corresponding author:** [yannick.colin@univ-rouen.fr](mailto:colin.yannick@gmail.com%20/)

**Keywords:** Gut bacterial composition and diversity; European chub; Acanthocephalans; Organic micropollutants; Intestinal parasite

**Figure S1. Rarefaction curves.** The curves indicate the observed number of bacterial Amplicon Sequence Variants (ASVs) detected within a sample.

**Figure S2. Relative abundance of the main bacterial phyla identified in fish gut bacterial communities.** The relative abundance of main bacterial phyla was estimated in controls (CTRL) and PAH-exposed fish (PAHs). The occurrence of the intestinal parasite *Pomphorhynchus sp*. was checked during dissection and then considered when analyzing the gut microbiota (i.e., infected and uninfected). The category “Others” regroups minor phyla (mean relative abundance < 0.5% of total reads) and unclassified reads. Each group tested contains a total of five samples. The values of the mean and the standard deviation corresponding to each phylum are summarized in Table S4.

**Figure S3. Relative abundance of the main bacterial phyla in gut bacterial communities.** The relative abundances of the phyla were compared between controls (n=5) and PAH-exposed chub (n=5), independently for uninfected (a) and infected chub (b). The color refers to the PAH exposure (gray: PAH-exposed and blue: controls). Pairwise Wilcoxon Mann-Whitney rank sum tests were used to compare the groups for each taxon. The *p*-values were adjusted with Benjamini-Hochberg corrections to correct for multiple testing (see Table S7 for details of adjusted *p*-values).

**Figure S4. Relative abundance of the main bacterial genera in gut bacterial communities.** The relative abundances of the genera were compared between controls (n=5) and PAH-exposed chub (n=5), independently for uninfected (a) and infected chub (b). The color refers to the PAHs exposure (gray: PAH-exposed and blue: controls). Pairwise Mann Whitney Wilcoxon tests were used to compare the groups for each taxon. The *p*-values were adjusted with Benjamini-Hochberg corrections to correct for multiple testing (see Table S8 for details of adjusted *p*-values).

**Table S1.** A table presenting the identity and parasite burden of the fish used to form the sample pools (3 fish per pool). These sample pools were subsequently used for the molecular analysis of chub microbiota. For each pool formed, the total biomass of gut tissue used for DNA extraction is indicated.

| **ID_Fish** | **[PAHs]** | **Parasite** | **Parasite burden** | | **ID_pool** | **Biomass used for DNA extraction (g)** |
| --- | --- | --- | --- | --- | --- | --- |
|  |  |  | **Per fish** | **Mean** |  |  |
| A08 ; A22 ; A28 | Control | Infected | 6 ;9 ;4 | **6.3** | F9 | 0.1557 |
| A11 ; A13 ; A19 | Control | Infected | 6 ;4 ;7 | **5.7** | G4 | 0.1676 |
| A34 ; A35 ; A37 | Control | Infected | 3 ;6 ;9 | **6.0** | G11 | 0.204 |
| A42 ; A45 ; A49 | Control | Infected | 4 ;5 ;4 | **4.3** | H3 | 0.2053 |
| A01 ; A04 ; A07 | Control | Infected | 5 ;3 ;6 | **4.7** | H6 | 0.101 |
| A03 ; A06 ; A09 | Control | Uninfected | - | **-** | G2 | 0.1236 |
| A27 ; A30 ; A31 | Control | Uninfected | - | **-** | G6 | 0.1868 |
| A29 ; A41 ; A43 | Control | Uninfected | - | **-** | G8 | 0.1529 |
| A12 ; A14 ; A15 | Control | Uninfected | - | **-** | H1 | 0.1781 |
| A20 ; A21 ; A24 | Control | Uninfected | - | **-** | H4 | 0.2408 |
| C26 ; C28 ; C34 | PAHs | Infected | 4 ;2 ;6 | **4.0** | G1 | 0.1013 |
| C11 ; C12 ; C16 | PAHs | Infected | 2 ;3 ;4 | **3.0** | G5 | 0.1483 |
| C17 ; C37 ; C40 | PAHs | Infected | 6 ;7 ;3 | **5.3** | G9 | 0.1975 |
| C42 ; C43 ; C45 | PAHs | Infected | 4 ;3 ;4 | **3.7** | G12 | 0.1878 |
| C01 ; C04 ; C07 | PAHs | Infected | 3 ;5 ;6 | **4.7** | H2 | 0.2116 |
| C21 ; C23 ; C25 | PAHs | Uninfected | - | **-** | F8 | 0.1289 |
| C29 ; C31 ; C32 | PAHs | Uninfected | - | **-** | G3 | 0.1943 |
| C35 ; C38 ; C40 | PAHs | Uninfected | - | **-** | G7 | 0.2248 |
| C46 ; C47 ; C48 | PAHs | Uninfected | - | **-** | G10 | 0.1226 |
| C05 ; C06 ; C20 | PAHs | Uninfected | - | **-** | H7 | 0.1218 |

**Table S2.** **Sequence statistics during processing in DADA2**

| **Samples** | **Input seq** | **Filtered seq** | **Denoised forward** | **Denoised reverse** | **Merged seq** | **Non chimera seq** | **Perc. retained** |
| --- | --- | --- | --- | --- | --- | --- | --- |
| **F8** | 75200 | 59068 | 58881 | 56498 | 52479 | 44404 | 59.0 |
| **F9** | 263212 | 211663 | 210541 | 198128 | 186626 | 162886 | 61.9 |
| **G1** | 230816 | 181669 | 180757 | 169123 | 158528 | 139051 | 60.2 |
| **G2** | 115894 | 89222 | 88682 | 86409 | 81473 | 75527 | 65.2 |
| **G3** | 105260 | 84504 | 84154 | 81045 | 76420 | 67784 | 64.4 |
| **G4** | 156162 | 127466 | 126812 | 120851 | 113917 | 104233 | 66.7 |
| **G5** | 190852 | 153068 | 151277 | 146159 | 135858 | 121733 | 63.8 |
| **G6** | 56236 | 43996 | 43816 | 42548 | 40190 | 37072 | 65.9 |
| **G7** | 59976 | 46161 | 45846 | 44948 | 42835 | 39762 | 66.3 |
| **G8** | 178488 | 141329 | 140226 | 136373 | 127009 | 119190 | 66.8 |
| **G9** | 97868 | 80194 | 79809 | 76671 | 72107 | 65742 | 67.2 |
| **G10** | 57543 | 45529 | 45396 | 43920 | 41184 | 38021 | 66.1 |
| **G11** | 324334 | 259544 | 258556 | 244724 | 229168 | 194652 | 60.0 |
| **G12** | 119653 | 96182 | 95704 | 91618 | 86558 | 81183 | 67.8 |
| **H1** | 49310 | 37379 | 37227 | 36133 | 33966 | 30845 | 62.6 |
| **H2** | 175890 | 139188 | 138582 | 132113 | 123940 | 109762 | 62.4 |
| **H3** | 276329 | 221405 | 220421 | 207816 | 196138 | 174036 | 63.0 |
| **H4** | 51021 | 39469 | 39258 | 38332 | 36623 | 34349 | 67.3 |
| **H6** | 360221 | 283472 | 282594 | 265987 | 249255 | 211230 | 58.6 |
| **H7** | 76274 | 59747 | 58625 | 56208 | 52616 | 46968 | 61.6 |
| **Total sum** | **3,020,539** | **2,400,255** | **2,387,164** | **2,275,604** | **2,136,890** | **1,898,430** |  |

**Table S3. Fish gut bacterial richness and diversity estimates.** Observed richness (Sobs) and Shannon-Weaver (H’) indexes were estimated in the controls (CTRL) and PAH-exposed fish (PAHs). Presented values are the mean and the standard deviation corresponding to each group.

|  | **Uninfected** | | | **Infected** | |  |
| --- | --- | --- | --- | --- | --- | --- |
|  | **CTRL** | **PAHs** | **Total** | **CTRL** | **PAHs** | **Total** |
| **Richness (Sobs)** | 1178 ± 258 | 909 ± 252 | **1044 ± 279** | 1205 ± 147 | 1149 ± 218 | **1177 ± 178** |
| **Shannon (H’)** | 4.16 ± 0.36 | 3.30 ± 0.9 | **3.73 ± 0.79** | 3.17 ± 0.22 | 3.28 ± 0.84 | **3.22 ± 0.58** |
| **Simpson** | 0.94 ± 0.03 | 0.82 ± 0.19 | **0.88 ± 0.14** | 0.77 ± 0.10 | 0.75 ± 0.15 | **0.76 ± 0.12** |

**Table S4. Relative abundance of the main bacterial phyla identified in fish gut bacterial communities.** The relative abundance of main bacterial phyla (mean ± sd) was estimated in the controls (CTRL) and PAH-exposed fish (PAHs). The parasitic status of the fish (i.e., I: infected, NI: uninfected) was checked during dissection and then taken into account when analyzing the gut microbiota.

|  | **Uninfected** | | **Infected** | |
| --- | --- | --- | --- | --- |
|  | **CTRL** | **PAHs** | **CTRL** | **PAHs** |
| ***Actinobacteriota*** | 23.59 ± 3.05 | 16.93 ± 13.29 | 1.80 ± 1.81 | 3.18 ± 2.83 |
| ***Alphaproteobacteria*** | 6.45 ± 4.2 | 28.19 ± 38.93 | 0.49 ± 0.22 | 6.07 ± 8.49 |
| ***Bacteroidota*** | 9.68 ± 11.93 | 3.29 ± 5.44 | 1.17 ± 2.39 | 3.92 ± 7.69 |
| ***Cyanobacteria*** | 1.81 ± 1.6 | 1.04 ± 2.03 | 0.05 ± 0.06 | 0.06 ± 0.04 |
| ***Firmicutes*** | 16.62 ± 9.81 | 29.97 ± 29.91 | 63.03 ± 21.21 | 56.16 ± 34.22 |
| ***Fusobacteriota*** | 0.72 ± 0.78 | 0.69 ± 0.81 | 22.32 ± 13.45 | 10.07 ± 13.39 |
| ***Gammaproteobacteria*** | 37.28 ± 19.16 | 17.44 ± 18.67 | 10.94 ± 16.74 | 18.53 ± 31.97 |

**Table S5. Summary of pairwise Mann Whitney Wilcoxon tests.** Pairwise Mann Whitney Wilcoxon tests were conducted for each main bacterial phylum to determine differences in relative abundance between uninfected (n=10) and Infected chub (n=10) and between the controls (CTRL, n=10) and PAH-exposed chub (PAHs, n=10) (see Figure 3). The p-values of the tests are presented in the table (p.value) and experiment-wise error was controlled using Bonferroni correction (p.adj). Significantly differentially abundant taxa were highlighted in bold.

| **Phylum** | **Uninfected (n=10) vs Infected (n=10)** | |
| --- | --- | --- |
|  | **p.value** | **p.adj** |
| *Gammaproteobacteria* | 0.1051 | 0.1051 |
| ***Fusobacteriota*** | **0.0052** | **0.0156** |
| ***Firmicutes*** | **0.0089** | **0.0156** |
| *Cyanobacteria* | 0.0433 | 0.0606 |
| *Bacteroidota* | 0.1051 | 0.1051 |
| ***Alphaproteobacteria*** | **0.0089** | **0.0156** |
| ***Actinobacteriota*** | **0.0003** | **0.0023** |
|  |  |  |
| **Phylum** | **CTRL (n=10) vs PAHs (n=10)** | |
|  | **p.value** | **p.adj** |
| *Gammaproteobacteria* | 0.6305 | 0.8827 |
| *Fusobacteriota* | 0.2475 | 0.5774 |
| *Firmicutes* | 0.9118 | 0.9118 |
| *Cyanobacteria* | 0.1903 | 0.5774 |
| *Bacteroidota* | 0.4813 | 0.8422 |
| *Alphaproteobacteria* | 0.2475 | 0.5774 |
| *Actinobacteriota* | 0.9118 | 0.9118 |

**Table S6. Summary of pairwise Mann Whitney Wilcoxon tests.** Pairwise Mann Whitney Wilcoxon tests were conducted for each main bacterial genus to determine differences in relative abundance between uninfected (n=10) and infected chub (n=10) and between the controls (CTRL, n=10) and PAH-exposed chub (PAHs, n=10) (see Figure 4). The p-values of the tests are presented in the table (p.value) and experiment-wise error was controlled using Bonferroni correction (p.adj). Significantly differentially abundant taxa were highlighted in bold.

| **Genus** | **Uninfected (n=10) vs Infected (n=10)** | |
| --- | --- | --- |
|  | **p.value** | **p.adj** |
| *(Gammaproteobacteria) Shewanella* | 0.4813 | 0.5769 |
| ***(Gammaproteobacteria) Pseudomonas*** | **0.0029** | **0.0069** |
| *(Gammaproteobacteria) Aeromonas* | 0.3930 | 0.5769 |
| ***(Fusobacteriota) Cetobacterium*** | **0.0052** | **0.0104** |
| ***(Firmicutes) Tyzzerella*** | **0.0001** | **0.0009** |
| *(Firmicutes) Enterococcus* | 0.3150 | 0.5400 |
| ***(Firmicutes) Candidatus_Bacilloplasma*** | **0.0021** | **0.0063** |
| *(Bacteroidota) Cloacibacterium* | 0.9705 | 0.9705 |
| *(Alphaproteobacteria) Rickettsia* | 0.5288 | 0.5769 |
| ***(Alphaproteobacteria) Neorickettsia*** | **0.0011** | **0.0042** |
| *(Actinobacteriota) Corynebacterium* | 0.4359 | 0.5769 |
| ***(Actinobacteriota) Aurantimicrobium*** | **0.0003** | **0.0019** |
|  |  |  |
| **Genus** | **CTRL (n=10) vs PAHs (n=10)** | |
|  | **p.value** | **p.adj** |
| *(Gammaproteobacteria) Shewanella* | 0.0232 | 0.1394 |
| *(Gammaproteobacteria) Pseudomonas* | 0.3527 | 0.6738 |
| *(Gammaproteobacteria) Aeromonas* | 0.3930 | 0.6738 |
| *(Fusobacteriota) Cetobacterium* | 0.2176 | 0.6527 |
| *(Firmicutes) Tyzzerella* | 0.5787 | 0.7717 |
| *(Firmicutes) Enterococcus* | 0.1051 | 0.4205 |
| *(Firmicutes) Candidatus_Bacilloplasma* | 0.3930 | 0.6738 |
| *(Bacteroidota) Cloacibacterium* | 0.9705 | 0.9705 |
| *(Alphaproteobacteria) Rickettsia* | 0.0232 | 0.1394 |
| *(Alphaproteobacteria) Neorickettsia* | 0.5288 | 0.7717 |
| *(Actinobacteriota) Corynebacterium* | 0.9118 | 0.9705 |
| *(Actinobacteriota) Aurantimicrobium* | 0.7959 | 0.9551 |

**Table S7. Summary of pairwise Mann Whitney Wilcoxon tests.** Pairwise Mann Whitney Wilcoxon tests were conducted for each main bacterial phylum to determine differences in relative abundance between the controls (CTRL, n=5) and PAHs-exposed chub (PAH, n=5), independently for parasite uninfected and infected chub (see Figure S3). The p-values of the tests are presented in the table (p.value) and experiment-wise error was controlled using Bonferroni correction (p.adj).

|  | **Uninfected** | |
| --- | --- | --- |
| **Phylum** | **CTRL (n=5) vs PAHs (n=5)** | |
|  | **p.value** | **p.adj** |
| *Gammaproteobacteria* | 0.2222 | 0.5185 |
| *Fusobacteriota* | 0.6905 | 0.6905 |
| *Firmicutes* | 0.6905 | 0.6905 |
| *Cyanobacteria* | 0.1508 | 0.5185 |
| *Bacteroidota* | 0.1508 | 0.5185 |
| *Alphaproteobacteria* | 0.6905 | 0.6905 |
| *Actinobacteriota* | 0.5476 | 0.6905 |
|  |  |  |
|  | **Infected** | |
| **Phylum** | **CTRL (n=5) vs PAHs (n=5)** | |
|  | **p.value** | **p.adj** |
| *Gammaproteobacteria* | 0.6905 | 0.9667 |
| *Fusobacteriota* | 0.2222 | 0.9583 |
| *Firmicutes* | 1.0000 | 1.0000 |
| *Cyanobacteria* | 0.8413 | 0.9815 |
| *Bacteroidota* | 0.5476 | 0.9583 |
| *Alphaproteobacteria* | 0.4206 | 0.9583 |
| *Actinobacteriota* | 0.5476 | 0.9583 |

**Table S8. Summary of pairwise Mann Whitney Wilcoxon tests.** Pairwise Mann Whitney Wilcoxon tests were conducted for each main bacterial genus to determine differences in relative abundance between the controls (CTRL, n=5) and PAHs-exposed chub (PAH, n=5), independently for parasite uninfected and infected chub (see Figure S4). The p-values of the tests are presented in the table (p.value) and experiment-wise error was controlled using Bonferroni correction (p.adj).

|  | **Uninfected** | |
| --- | --- | --- |
| **Genus** | **CTRL (n=5) vs PAHs (n=5)** | |
|  | **p.value** | **p.adj** |
| *(Gammaproteobacteria) Shewanella* | 0.0317 | 0.1905 |
| *(Gammaproteobacteria) Pseudomonas* | 1.0000 | 1.0000 |
| *(Gammaproteobacteria) Aeromonas* | 0.3095 | 0.7211 |
| *(Fusobacteriota) Cetobacterium* | 0.5476 | 0.8214 |
| *(Firmicutes) Tyzzerella* | 0.4206 | 0.7211 |
| *(Firmicutes) Enterococcus* | 0.4206 | 0.7211 |
| *(Firmicutes) Candidatus_Bacilloplasma* | 0.1508 | 0.6032 |
| *(Bacteroidota) Cloacibacterium* | 0.8413 | 1.0000 |
| *(Alphaproteobacteria) Rickettsia* | 0.0317 | 0.1905 |
| *(Alphaproteobacteria) Neorickettsia* | 0.3095 | 0.7211 |
| *(Actinobacteriota) Corynebacterium* | 0.6905 | 0.9206 |
| *(Actinobacteriota) Aurantimicrobium* | 1.0000 | 1.0000 |
|  |  |  |
|  | **Infected** | |
| **Genus** | **CTRL (n=5) vs PAHs (n=5)** | |
|  | **p.value** | **p.adj** |
| *(Gammaproteobacteria) Shewanella* | 0.8413 | 1.0000 |
| *(Gammaproteobacteria) Pseudomonas* | 0.1508 | 0.8889 |
| *(Gammaproteobacteria) Aeromonas* | 0.8413 | 1.0000 |
| *(Fusobacteriota) Cetobacterium* | 0.2222 | 0.8889 |
| *(Firmicutes) Tyzzerella* | 0.4206 | 1.0000 |
| *(Firmicutes) Enterococcus* | 0.2222 | 0.8889 |
| *(Firmicutes) Candidatus_Bacilloplasma* | 0.6905 | 1.0000 |
| *(Bacteroidota) Cloacibacterium* | 0.8413 | 1.0000 |
| *(Alphaproteobacteria) Rickettsia* | 0.3095 | 0.9286 |
| *(Alphaproteobacteria) Neorickettsia* | 1.0000 | 1.0000 |
| *(Actinobacteriota) Corynebacterium* | 0.8413 | 1.0000 |
| *(Actinobacteriota) Aurantimicrobium* | 1.0000 | 1.0000 |
